# The distribution and prognosis of anomalous coronary arteries identified by cardiovascular magnetic resonance: 15 year experience from two tertiary centres

**DOI:** 10.1186/1532-429X-16-34

**Published:** 2014-05-20

**Authors:** David P Ripley, Ansuman Saha, Albert Teis, Akhlaque Uddin, Petra Bijsterveld, Ananth Kidambi, Adam K McDiarmid, Mohan Sivananthan, Sven Plein, Dudley J Pennell, John P Greenwood

**Affiliations:** 1 Multidisciplinary Cardiovascular Research Centre (MCRC) & Leeds Institute of Genetics, Health and Therapeutics, University of Leeds, Leeds, UK; 2 Leeds Teaching Hospitals NHS Trust, Leeds General Infirmary, Leeds, UK; 3 NIHR Cardiovascular Biomedical Research Unit Royal Brompton Hospital, London, UK

**Keywords:** Coronary vessel anomalies, Cardiovascular magnetic resonance, Prognosis

## Abstract

**Background:**

Aberrant coronary arteries represent a diverse group of congenital disorders. Post-mortem studies reveal a high risk of exercise-related sudden cardiac death in those with an anomalous coronary artery originating from the opposite sinus of Valsalva (ACAOS) with an inter-arterial course. There is little documentation of lifetime history and long-term follow-up of patients with coronary artery anomalies.

**Methods:**

Patients with anomalous coronary arteries undergoing cardiovascular magnetic resonance over a 15-year period were identified and classified by anatomy and course. Medical records were reviewed for major adverse cardiovascular events (MACE). Revascularisation or myocardial infarction counted only if occurring in the distribution of the anomalous artery.

**Results:**

Consecutive patients with coronary artery anomalies were retrospectively identified (n = 172). Median follow-up time was 4.3 years (IQR 2.5–7.8, maximum 15.6). 116 patients had ACAOS of which 64 (55%) had an inter-arterial course (IAC) and 52 (45%) did not. During follow up 110 ACAOS patients were alive, 5 died and 1 lost to follow-up.

ACAOS patients experienced 58 MACE events (5 cardiovascular deaths, 5 PCI, 24 CABG and 24 had myocardial infarction). 47 MACE events occurred in ACAOS with IAC and 11 in those without (p < 0.0001), the statistical difference driven by surgical revascularisation and myocardial infarction.

**Conclusions:**

In life, patients with an anomalous coronary artery originating from the opposite sinus of Valsalva taking an IAC have higher rates of both myocardial infarction and surgical revascularisation during long-term follow up, compared to those without IAC.

## Background

Aberrant coronary arteries represent a diverse group of congenital disorders which affect between 0.3 to 1.3% of the unselected general population undergoing cardiac catheterisation [1,2]. They are increasingly recognised due to the expansion of advanced non-invasive cardiovascular imaging techniques including computed tomography (CT) imaging and cardiovascular magnetic resonance (CMR).

The majority of coronary anomalies go unrecognised and have no clinical significance, although a minority have been documented to have increased risk of myocardial infarction and ischaemia, congestive heart failure and sudden cardiac death (SCD). Congenital coronary abnormalities are the second leading cause of SCD in the young exceeded only by hypertrophic cardiomyopathy (HCM) [3]. Those with the highest risk include individuals with an anomalous coronary artery originating from the opposite sinus of Valsalva (ACAOS) with this risk higher in athletes than non-athletic persons [4]. Furthermore post mortem studies suggest this risk is limited to those ACAOS which take an inter-arterial course (IAC) between the aorta and pulmonary artery (PA), and this has been documented in both anomalous origins of the right coronary artery from the left coronary sinus (right-ACAOS) and in anomalous origins of the left coronary artery from the right coronary sinus (left-ACAOS); the latter appears to carry a higher risk of SCD [5-7]. Other anatomical features which are associated with high risk include intra-mural course, slit-like orifice and angulated take-off which are all thought to contribute to myocardial ischaemia [8].

Surgical intervention in patients with ACAOS, such as re-implantation of the anomalous vessel into the correct coronary sinus, unroofing of the intra-mural course or coronary artery bypass grafting may be performed which in theory prevents ischaemia and/or SCD. There are favourable early outcome data from surgical intervention [9,10] although little data regarding long term follow-up for late complications and outcomes. Although Krasuski et al. demonstrated contrasting results showing similar mortality rates between medically and surgically treated groups with ACAOS [10].

There is little published evidence describing the long term history and long-term follow-up of anomalous coronary arteries in life. The aim of this study was to demonstrate the distribution of anomalous coronary arteries referred for CMR and to document their long-term clinical outcome with respect to major adverse cardiovascular event (MACE) rates. A secondary aim was to confirm whether or not the anatomical course of anomalous coronary arteries conferred differential risk.

## Methods

This study was approved by a regional research ethics committee and the National Information Governance Board for Health and Social Care (NIGB, section 251). Databases from two large UK cardiovascular MR centres were reviewed (Leeds General Infirmary and the Royal Brompton Hospital, London). All patients who were referred for clinically indicated CMR over a 15 year period (1995 to 2010) with anomalous coronary arteries identified on those scans were recorded. All CMR scans were performed with a 1.5 Tesla Philips Intera CV scanner (Philips Healthcare, Best, The Netherlands) or a 1.5 Tesla Siemens Sonata or Avanto scanner (Siemens Healthcare, Erlangen, Germany).

All CMR images were recalled and independently retrospectively reviewed. The anomalous coronary arteries were classified according to their anatomy and course, following strict criteria (Table 1).

**Table 1:** Classification of anomalous coronary arteries*

	
1. Origin of both RCA and LMS (separate origins) from the right aortic sinus	
	1a. Course of anomalous LMS between aorta and pulmonary artery (PA)
	1b. Course of anomalous LMS *not* between aorta and PA
2. Origin of both coronary arteries (separate origins) from the left aortic sinus	
	2a. Course of anomalous RCA between aorta and PA
	2b. Course of anomalous RCA *not* between aorta and PA
3. Anomalous origin of the circumflex coronary artery from the right aortic sinus	
	3a. Course of anomalous LCx between aorta and PA
	3b. Course of anomalous LCx *not* between aorta and PA
4. Anomalous origin of the left anterior descending artery from the right aortic sinus	
	4a. Course of anomalous LAD between aorta and PA
	4b. Course of anomalous LAD *not* between aorta and PA
5. Single coronary artery (common origin)	
	5a. Course of anomalous coronary artery between aorta and PA
	5b. Course of anomalous coronary artery *not* between aorta and PA
6. Anomalous origin or communication of a coronary artery with a cardiac chamber or major thoracic vessel	
	6a. Abnormal origin from the pulmonary artery or one of its major arterial branches
	6b. Abnormal origin from the aorta or one of its major arterial branches
	6c. Abnormal communication of a coronary artery with a cardiac chamber or major thoracic vessel (fistula).
7. Miscellaneous/unclassified	

*Adapted from Angelini Circulation 2002 [11] and Wilkins Texas Heart Institute Journal 1988 [12].

Both the electronic and paper records of all patients were reviewed for MACE. Confirmation of events was further clarified from the general practitioner records. Their vital status (dead or alive) was ascertained from National Health Service Spine electronic record and if the patient was dead, the cause of death retrieved from the Office of National Statistics. MACE was defined as cardiovascular death, myocardial infarction in the distribution of the anomalous coronary artery, revascularisation in the distribution of the anomalous coronary artery or aborted SCD. Definition and adjudication of MACE was performed blinded to the coronary anomaly classification.

### CMR pulse sequences

CMR pulse sequences were performed using a protocol which included a contemporary (at the time of patient study) coronary magnetic resonance angiography pulse sequence. A low-resolution coronary survey scan was initially performed during free breathing, using a respiratory navigator. For a targeted approach CMR was performed as follows: for the right coronary artery (RCA) double-oblique three dimensional volume along the long-axis of the RCA; for the left coronary system a double-oblique transverse three dimensional volume centred on the left main stem through the proximal left anterior descending and left circumflex arteries. For anomalous coronaries separate targeted views were typically acquired using a 3 point planning technique. 3-Dimensional targeted volumes were acquired using a three-dimensional gradient-echo sequence. Typical pulse sequence parameters: TE 2.2 ms, TR 7.7 ms, flip angle 25°, T2 and fat saturation pre-pulses, matrix 512 × 360, field of view 360 mm, 20 slices with slice thickness of 1.5 mm, acquired voxel size of 0.7 × 1.0 mm (reconstructed to 0.7 × 0.7 mm). Full details previously reported [13].

More recent techniques included a three dimensional whole heart acquisition. For this, timing of the diastolic coronary rest period was estimated from a high temporal resolution four-chamber free breathing cine scan. Three dimensional whole heart coronary MR angiography was acquired using a balanced SSFP sequence and a respiratory navigator to compensate for respiratory motion during free breathing. Typical pulse sequence parameters: TE 2.3 ms, TR 4.6 ms, flip angle 100°, T2 and fat saturation pre-pulses, duration of acquisition up to 120 ms per R-R interval (determined by length of diastolic rest period), matrix 304 × 304, field of view 320–460 mm, slice thickness 0.9 mm, 100–120 slices as required, acquired in-plane voxel size of 1.0 × 1.0 mm (reconstructed to 0.5 × 0.5 mm) [14].

### Statistics

Data analysis was performed using SSPS 17.0 for Windows (SPSS, Chicago, Illinois, USA). Data are presented as mean ± SD or median (IQR) as appropriate. Ordinal data were compared using the Mann–Whitney U and *χ*^2^ tests. Two-tailed testing with a probability value of ≤0.05 was considered statistically significant. Kaplan-Meier curves were used to estimate the distribution of time to first cardiac event and differences between time to first event curves compared with the log-rank test.

## Results

**Figure 1:**
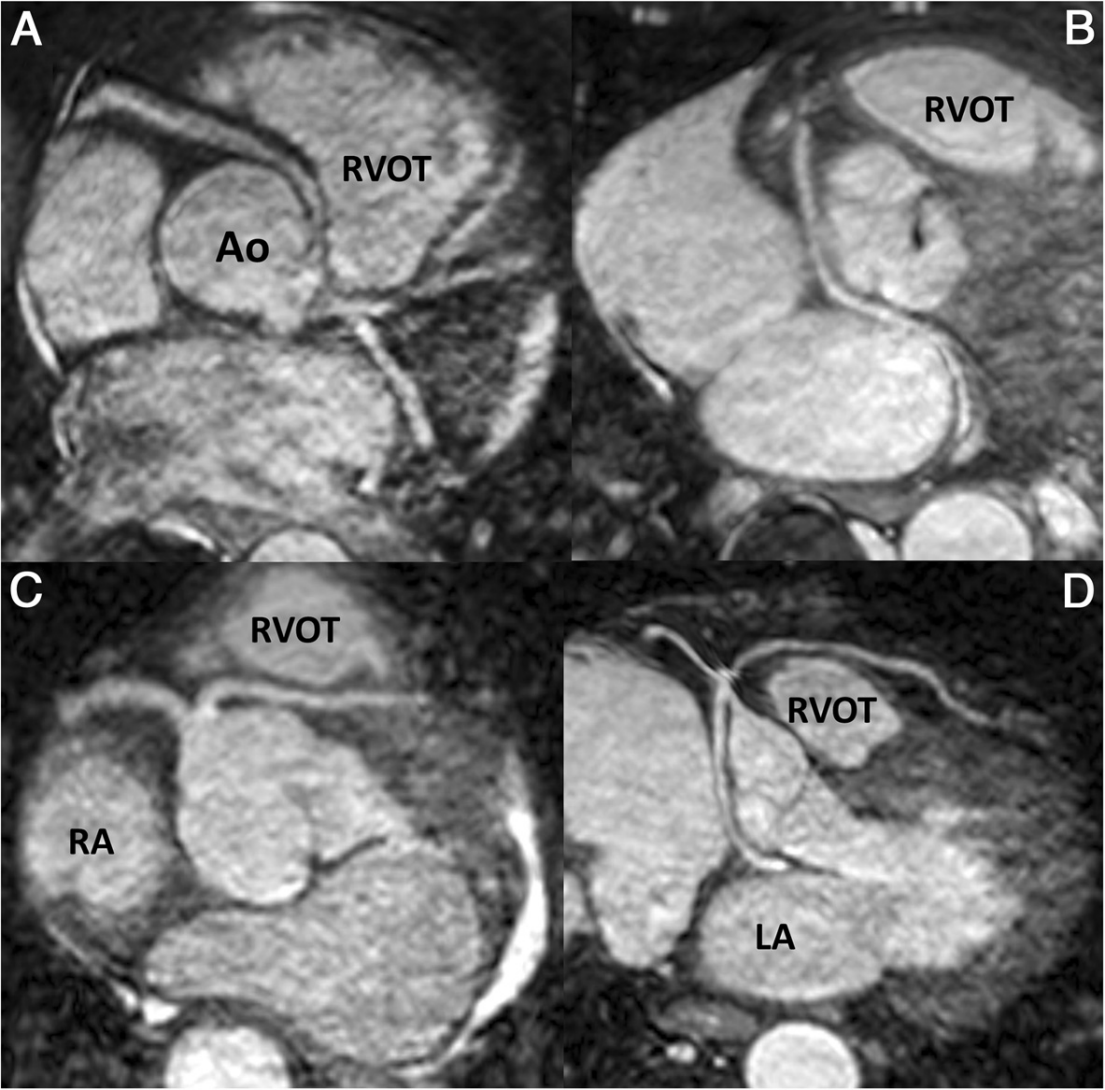
**Examples of several different anomalous coronary arteries imaged by CMR: (A)** Right coronary artery originating from the left coronary cusp passing anteriorly (class 2a), **(B)** Circumflex artery arising from the right coronary cusp (RCC) passing posteriorly (class 3b), **(C)** Left anterior descending (LAD) artery from the RCC anteriorly passing between the RVOT and aorta (class 4a) and **(D)** single ostium coronary system arising from the RCC with a retro-aortic circumflex artery and a pre-pulmonic LAD (class 5b). Images reconstructed using SoapBubble^TM^ software (Philips Medical Systems). Ao - Aorta; RVOT – Right Ventricular Outflow Tract; RA – Right Atrium; LA – Left Atrium.

In total, 59,844 CMR scans were performed between March 1995 and April 2010 at the two CMR institutions. 172 consecutive patients (0.3%) with coronary artery anomalies were retrospectively identified (90 and 82 from each centre) with a median age of 54 years (range 1–85; IQR 40–64). The median follow-up time was 4.3 years (IQR 2.5–7.8) with a maximum of 15.6 years. At the time of follow-up, 160 patients were alive (93%), 10 deceased (6%) and 2 lost to follow-up. The distribution of classified coronary anomalies are detailed in Table 2 and a selection of coronary anomalies imaged by CMR are shown in Figure 1. There were no cases where the anatomy could not be determined.

**Table 2:** Distribution of the coronary anomalies in the joint CMR Databases (n = 172)

**Classification**	**Number**	**Age (median, range)**
1a	13	58, 8 – 73
1b	8	36, 1 – 73
2a	34	55, 13 – 78
2b	0	
3a	0	
3b	33	58, 25 – 78
4a	4	56, 30 – 70
4b	6	19, 15 – 41
5a	13	60, 29 – 85
5b	5	70, 34 – 80
6a	11	36, 15 – 64
6b	2	53, 47 – 59
6c	18	56, 18 – 76
7	25	46, 8 – 78

Of the 172 patients, 116 had ACAOS (61 and 55 from each centre) with a median follow-up of 4.6 years. Of these, 110 were alive (95%), 5 deceased (4.3%) and 1 lost to follow-up (1%). 21 patients had a separate right coronary artery (RCA) and left main stem (LMS) from the right coronary sinus of which 13 had an IAC of the LMS between the aorta and PA. Separate origins of both coronary arteries from the left aortic sinus all of which took an IAC were present in 34 patients. An anomalous circumflex from the opposite sinus occurred in 33 patients none of which took an IAC. An anomalous left anterior descending artery from the opposite sinus was present in 10 patients, with 4 following an IAC route. Finally, 18 had a single coronary artery of which 13 passed inter-arterially. Therefore of the 116 patients with ACAOS, 64 patients (55%) had an inter-arterial course and 52 (45%) without an inter-arterial course (Table 3). There was no statistical difference in the age of the two ACAOS populations (with or without IAC) (P = 0.63).

**Table 3:** Distribution of anomalous coronary arteries originating from the opposite sinus with and without inter arterial course

	**Number**	**Age (median, range)**
Total ACAOS	116	54, 1 – 85
- ACAOS with inter-arterial course	64	56, 8 – 85
- ACAOS without inter-arterial course	52	53, 1 – 80

ACAOS = anomalous coronary artery originating from the opposite sinus of Valsalva.

Of those patients with ACAOS there were in total 58 MACE (5 cardiovascular deaths, 5 percutaneous coronary interventions (PCI) to the anomalous artery, 24 underwent surgical revascularisation to the anomalous territory and 24 had a documented myocardial infarction in the anomalous coronary artery territory). All PCI was performed for coronary artery disease with coronary stenosis visually measuring > 70% luminal stenosis. All myocardial infarctions were spontaneous related to atherosclerotic plaque (type 1) [15]. The mortality rate from cardiovascular causes was 4.3% (5 cardiovascular deaths in 116 ACAOS patients) during the median follow up period of 4.6 years. Of the 5 deaths 4 were from a documented myocardial infarction (all with a rise of cardiac biomarkers and ECG changes) and one death from chronic heart failure secondary to ischaemic heart disease.

**Figure 2:**
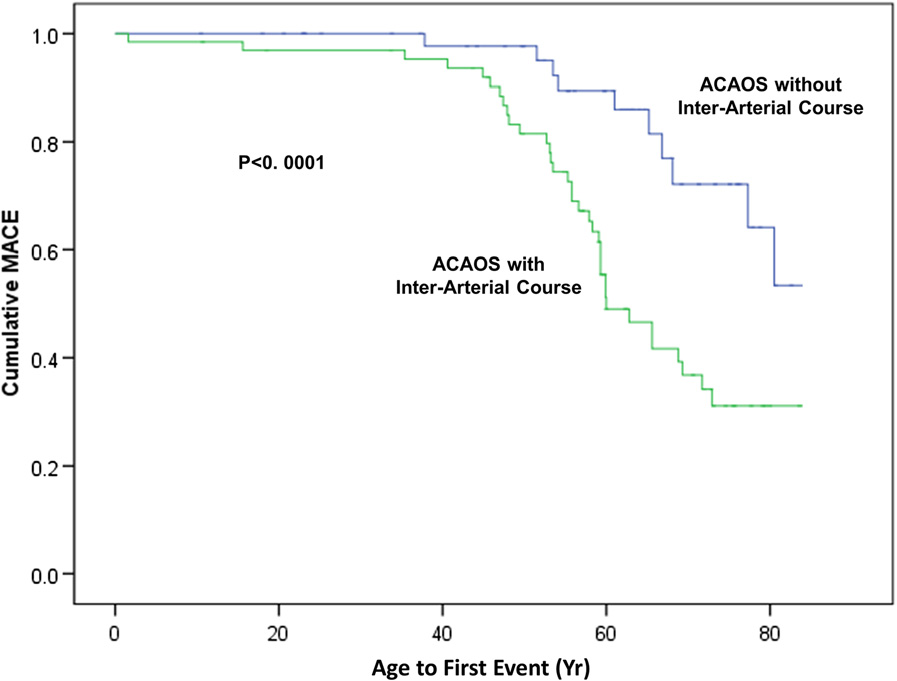
Kaplan-Meier curves showing the age at which the first major adverse cardiovascular event occurred, demonstrating a significant difference (log rank test, p < 0.0001) between those anomalous coronary arteries from the opposite sinus (ACAOS) with an inter-arterial course (IAC) and those without IAC.

47 MACE occurred in patients with ACAOS and an IAC and 11 in those without an IAC (p < 0.0001). There was significantly higher risk of both surgical revascularisation and myocardial infarction in the IAC group, with similar numbers of percutaneous revascularisation and cardiovascular deaths (Table 4). Age of first MACE was lower in those coronary abnormalities with an IAC (p < 0.0001) (Figure 2). The distribution of major cardiovascular events by coronary anomaly is presented in Table 5.

**Table 4:** Major Adverse Cardiovascular Events in patients with an anomalous coronary artery originating from the opposite sinus of Valsalva (ACAOS) (n = 116)

	**ACAOS with IAC (n = 64)**	**ACAOS without IAC (n = 52)**	**P value**
**Cardiovascular Deaths, n**	3	2	NS
**PCI, n**	3	2	NS
**Surgical revascularisation, n**	23	1	P < 0.0001
**Myocardial Infarction, n**	18	6	P < 0.05

**Table 5:** Major Adverse Cardiovascular Events in patients by coronary anomaly in those patients with anomalous coronary artery originating from the opposite sinus of Valsalva (ACAOS)

**Classification**	**Number**	**Major Adverse Cardiovascular Event**				**Total MACE**
		**CV Death**	**PCI**	**Surgical revascularisation**	**MI**	
1a	13	0	1	7	5	13
1b	8	0	1	0	2	3
2a	34	1	1	12	6	20
2b	0	0	0	0	0	0
3a	0	0	0	0	0	0
3b	33	1	1	1	4	7
4a	4	0	0	0	2	2
4b	6	0	0	0	0	0
5a	13	2	1	4	5	12
5b	5	1	0	0	0	1

CV death – Cardiovascular death; PCI – Percutaneous coronary intervention; MI – myocardial infarction.

## Discussion

This study has shown the distribution of coronary artery anomalies imaged by CMR in two large tertiary cardiac centres over the previous 15 years. Our study demonstrates the prognostic significance of an inter-arterial course for an anomalous coronary artery originating from the opposite sinus of Valsalva (ACAOS) passing between the aorta and pulmonary artery. We have also shown significantly higher myocardial infarction and surgical revascularisation rates for this group of anomalous arteries in those patients referred for CMR, over a median 4.6 year follow-up period.

Invasive X-ray angiography has traditionally been the diagnostic imaging test to identify coronary anomalies however the presence of an anomalous vessel may only be suspected after the procedure (e.g. after unsuccessful catheterisation). CMR has a number of advantages for diagnosing coronary artery anomalies. The non-invasive technique does not require contrast agents or ionising radiation, which is an important consideration particularly in children and young adults. The dose and stochastic effects of X-ray radiation are frequently misjudged [16] and the risk of developing a solid tumour is estimated at 1:2500 diagnostic coronary angiographic procedures [17,18]. Furthermore CMR clarifies the spatial relationship of these arteries with respect to the aorta and the pulmonary artery, which we and others have demonstrated confers differential risk; this is also important in surgical planning. The application of CMR for the detection and documentation of the origin and course of coronary anomalies is well established [19-22] and as such CMR is a recognised class I indication in international guidelines [23,24].

Anomalous coronary arteries cover a wide range of conditions the majority of which have no clinical significance [25]. Post mortem studies from both the military and in young athletes have shown that ACAOS is associated with a higher risk of SCD. The risk is higher in athletes with ACAOS, representing a 79 times higher relative risk than in non-athletic individuals [4] which is limited to those ACAOS taking an inter-arterial course between the aorta and main pulmonary artery. In one 25 year review of 126 non-traumatic military deaths, a high proportion of deaths (33%) were attributable to ACAOS and it was a more common cause of death than both myocarditis and hypertrophic cardiomyopathy [26]. In this review, the risk was exclusively limited to only those with a left-ACAOS (anomalous origination of the left coronary artery from the right sinus of Valsalva [27]) passing inter-arterially. However, there is also evidence that right-ACAOS (anomalous origination of the right coronary artery from the left sinus of Valsalva) may cause SCD, with one registry in athletes attributing 27 deaths to ACAOS (4 of which were a right coronary artery from the left coronary sinus) [28]. This registry also confirmed the relationship with exertion, detailing that all deaths occurred either during or shortly after exercise [28]. The prognostic risk of right-ACAOS was further corroborated by a contemporary review of consecutive patients referred for CT coronary angiography over a 4 year period, demonstrating malignant features which interestingly in this series were exclusive to right-ACAOS [29]. Those high risk features of an intramural inter-arterial course and severe lateral compression of the proximal vessel have been shown to have a clear relationship with ventricular fibrillation [30].

Whilst there is evidence that ACAOS is associated with SCD, there are little data regarding myocardial infarction and rates of revascularisation in the anomalous artery territory. Chaitman *et al*. [31] demonstrated the existence of coronary atheroma in 6 out of 17 patients (35%) with ACAOS, significantly higher than the 8% documented in a normal population [32]. Furthermore a history of MI and in patients with ACAOS with IAC and non-atherosclerotic arteries has been documented [31]. Although one long term follow-up of 87 patients with right-ACAOS and an inter-arterial course showed only 3 myocardial infarctions (3.4%) over a 2.5 year follow-up [33] significantly lower than 15% we observe over 4.3 years in this sub-group (and 28% overall in our cohort). There are only a few published cases showing myocardial infarction in the anomalous territory.

The mechanism of myocardial infarction and SCD in ACAOS is not clear. The risk from ACAOS has been traditionally thought to be related directly to the haemodynamic significance of the inter-arterial course of the anomalous vessel, with SCD and myocardial infarction related to rising pressure in both aorta and pulmonary artery during exercise causing a scissoring or kinking of the vessel [34]. Angelini, however, has since developed a theory of intussusception, eloquently demonstrating with intravascular ultrasound (IVUS) that the main mechanism of ischaemia originates with hypoplasia and lateral luminal compression in the proximal vessel, which runs intramurally at the aortic root whilst crossing the aorto-pulmonary septum and demonstrated worsening of the proximal narrowing during physiological stress testing [35,36]. An extramural course has never been observed in those ACAOS with an inter-arterial course [25] however an intramural course has been demonstrated in some ACAOS without an inter-arterial course [25,27].

The location of the anomalous vessel in relation to the pulmonary trunk is therefore important and one long term follow up of right-ACAOS with inter-arterial course diagnosed by CT demonstrated higher MACE in those with a high inter-arterial course (RCA ostium located above the pulmonary valve) as opposed to low inter-arterial course (RCA ostium below pulmonary valve) and therefore hypothesised that the high inter-arterial course is more susceptible to compression between the aortic root and main pulmonary artery during systole [33]. The difference in MACE in this study was driven by unstable angina admissions and there was no statistical difference between myocardial infarction and cardiovascular death in both groups [33].

Krasuski *et al.*[10]. followed-up 301 patients with ACAOS (54 with an IAC) and demonstrated that ACAOS with an inter-arterial course were almost twice as likely to undergo surgical intervention and showed a higher rate of significant coronary artery disease in the distribution of the anomalous vessel (50% vs. 25%, p = 0.02) in those undergoing surgical intervention. Those undergoing surgery were also more likely to have had a myocardial infarction and an abnormal cardiac stress test [10]. They did not however, show any difference in the incidence of myocardial infarction between those with or without IAC, which is contrary to our data. Our study demonstrates a significantly higher rate of myocardial infarction in the territory of the anomalous coronary artery with 18 events in 64 ACAOS with IAC and only 6 in 52 ACAOS without IAC (p < 0.05). The lack of significance in myocardial infarction rates in the study by Krasuski may be due to the preference for early surgical intervention with 52% of patients with an inter-arterial course undergoing cardiac surgery, and after the year 2000, a 4:1 ratio of surgical versus medical management.

Krasuski also demonstrated a substantially higher mortality rate of 44% over a mean 9.2 year follow-up which is in contrast to the 4.3% mortality rate over 4.6 years in our study. Despite the high mortality rate they showed no difference in long term survival between those with or without IAC, which accords with our results, although contradicts the data from post-mortem studies. This lack of significance is unlikely to be due to the size of our cohort (n = 116) as this is higher than the population in many autopsy studies which reveal a statistical difference between the ACAOS with or without IAC. This may, however, reflect the limited number of young patients in our cohort with only 11% under the age of 30 years and a median age of 54 years. The under 30 age group in particular have been shown to have a significantly higher risk of SCD and exercise related death [8,37].

### Limitations

This is a retrospective review of a selected population of patients referred to tertiary CMR centres for documentation of their coronary anatomy and therefore subject to referral bias. It is recognised that all cardiovascular MR scans performed showing coronary artery abnormalities may not have been identified, for example some those with complex adult congenital heart disease and incidental coronary anomalies could have been missed; we were careful to include all those with anomalous coronaries as the primary referral indication for CMR. The mean age of the patients was 54 years and therefore does not include many of the younger high risk patients who may have already died from SCD. We were unable to fully document referral indication, cardiovascular risk profile, co-morbidities and medication which may have influenced patient outcomes. Also, invasive angiography was not mandated in all to confirm the presence or absence of epicardial coronary artery disease. However we were careful to focus on hard cardiovascular events that could easily be validated. We were also meticulous in determining that myocardial infarction and revascularisation had to be related to the aberrant vessel in order to count as MACE for the purpose of our study. Whilst it is recognised that surgical revascularisation may have been performed for primary prevention on knowledge of the coronary anatomy in selected cases, we note the very large difference in surgical revascularisation between ACAOS with IAC (23, 36%) and those without (1, 2%). Furthermore there remained a significant difference in the number of myocardial infarctions (18 vs. 6) between the ACAOS with and without IAC.

## Conclusion

In conclusion, we have shown distribution of anomalous coronary arteries imaged by CMR over a 15 year period from two large tertiary cardiac centres. This represents one of the largest series of anomalous coronary arteries, demonstrating that in life, patients with an anomalous coronary artery originating from the opposite sinus of Valsalva taking an inter-arterial course have higher rates of both myocardial infarction and surgical revascularisation during long-term follow up, compared to those without IAC.

## Consent

Written informed consent was obtained from the patient for the publication of this report and any accompanying images.

## Abbreviations

ACAOS: Anomalous coronary artery originating from the opposite sinus of Valsalva; CMR: Cardiovascular magnetic resonance; CT: Computed tomography; HCM: Hypertrophic cardiomyopathy; IAC: Inter-arterial course; IVUS: Intravascular ultrasound; LMS: Left main stem; MACE: Major adverse cardiovascular events; PA: Pulmonary artery; RCA: Right coronary artery; RVOT: Right ventricular outflow tract; SCD: Sudden cardiac death.

## Competing interest

The authors declare that they have no competing interest.

## Authors’ contributions

DPR: Design, analysis and interpretation of data, drafting of the manuscript; AS: Analysis and interpretation of data; AT: Analysis and interpretation of data; AU: Interpretation of data, critical and intellectual revision of manuscript; PB: Analysis and interpretation of data; AK: Critical and intellectual revision of manuscript; AKM: Critical and intellectual revision of manuscript; MS: Critical and intellectual revision of manuscript; SP: Critical and intellectual revision of manuscript; DJP: Conception and design, critical and intellectual revision of manuscript. JPG: Conception and design, interpretation of data, drafting of manuscript. All authors have given approval of this manuscript for publication.
